# Local Knowledge and Conservation Priorities of Medicinal Plants near a Protected Area in Brazil

**DOI:** 10.1155/2019/8275084

**Published:** 2019-02-03

**Authors:** Noelia Ferreira da Silva, Natalia Hanazaki, Ulysses Paulino Albuquerque, Juliana Loureiro Almeida Campos, Ivanilda Soares Feitosa, Elcida de Lima Araújo

**Affiliations:** ^1^Departamento de Biologia, Programa de Pós-Graduação em Botânica, Universidade Federal Rural de Pernambuco, Rua Dom Manoel de Medeiros, S/N, Dois Irmãos, 52171-900 Recife, PE, Brazil; ^2^Departamento de Ecologia e Zoologia (ECZ), Centro de Ciências Biológicas (CCB), Universidade Federal de Santa Catarina, Campus Universitário, Cidade Universitária, 88040-900 Florianópolis, SC, Brazil; ^3^Laboratório de Ecologia e Evolução de Sistemas Socioecológicos, Departamento de Botânica, Centro de Biociências, Universidade Federal de Pernambuco, Cidade Universitária, 50670-901 Recife, PE, Brazil

## Abstract

We investigated the influence of socioeconomic factors (age, gender, and occupation) on the local knowledge of medicinal plants in the Araripe National Forest, Brazil, and the priority of conservation of the species as perceived by people. Additionally, priority species for in situ conservation were identified by calculating conservation priority (CP). Initially, free lists were developed with 152 informants in order to identify the plants known and used by them. Based on the most cited plants in these lists, a salience analysis was performed to identify the ten most prominent tree species. In a second moment, through a participatory workshop, these ten species were classified by the perception of local experts as to their environmental availability and intensity of exploitation. Then, the population size of the forest plant species was quantified through a phytosociological sampling and the conservation priority index (CP) of the species was calculated. A total of 214 ethnospecies were cited by the informants, which were identified in 167 species. Local knowledge was influenced by socioeconomic factors, with positive correlation between age and local knowledge and difference in knowledge among professions. Among the ten most prominent tree species in terms of their medicinal importance,* Hancornia speciosa* was highlighted as a priority for conservation in the experts' perception because it has low environmental availability and a high exploitation rate. The ten species were ordered by the CP differently from the ordering made by the local experts' perception, indicating that people's perception of species conservation status may not correspond to the actual situation in which they are found in the forests. Conservationist measures based on the perception of informants need complementary ecological studies on the species accessed.

## 1. Introduction 

The collection and use of medicinal plants, although common practices in the different cultures of the world [1–3], may bring challenges for the conservation of the medicinal resource used. This is particularly true when there is a significant reduction in the population size of the exploited species, leading it to a risk of local extinction [4].

The local communities that use the medicinal resource are the first to realize its availability reduction and are, therefore, holders of a knowledge of expressive importance to determine the priority species for conservation, as well as for the elaboration of strategies that allow the sustainability of exploitation [5].

However, communities may differ among localities because of their socioeconomic characteristics, and some studies have shown that age, gender, and profession may influence people's knowledge of resource use [6–11]. This fact has indicated that the socioeconomic profile of human populations needs to be considered in the recovery of the perception of the local populations and in the conception of strategies aimed at the conservation of the exploited resource.

Among the strategies adopted by governments is the creation of conservation units [12], which often incorporate restrictive measures in order to introduce the sustainable use of resources, prohibiting the use of target species of intense extractivism in the region [13]. This decision, despite having a conservationist intent, often generates local problems and conflicts by not considering the perception and knowledge of the local populations about the practices of collection and use of the medicinal resource.

Accordingly, some studies report that forest resources continue to be used by the populations around conservation units, regardless of whether they are full protection or sustainable use [8, 14], especially when the populations have low purchasing power.

As an example, people that live in the vicinity of the Araripe National Forest (FLONA), a full protection conservation unit in the Northeastern region of Brazil, collect nontimber forest resources [7, 8] and wood [15] from the FLONA Araripe. Except for the collection of the fruits of pequi (*Caryocar coriaceum* Wittm.), all other resources are exploited clandestinely, that is, without authorization from the management of the Conservation Unit, which can be very negative for the conservation of some species.

Among the uses of the FLONA Araripe plant resources, we can highlight the medicinal use, since many people living in the surroundings of the protected forest have low income and need to make use of the plants to cure their diseases [7–9]. Therefore, based on the above, our expectation is that the local knowledge of these people on medicinal plants, considering their socioeconomic characteristics, allows identifying species that should be considered priorities for conservation. Evidencing these species is of fundamental importance for the establishment of measures by conservation units managers, being one of the necessary steps to minimize existing conflicts in socioecological systems regarding the use and conservation of forest resources.

Specifically, this study seeks to answer the following questions: are there differences in the total number of medicinal plants that are known and used? Is the number of plants cited influenced by the gender, age, and profession of the informant? Are there any medicinal woody species that need conservation practices in the region? Which parts of the plants stand out in medicinal use? Is there a difference between the local perception and the calculation of the conservation priority in relation to the conservation status of the species?

## 2. Material and Methods

### 2.1. Study Area

The study was executed in the rural community of Macaúba (S 7° 21′ 10.2′′ W 39° 24′ 12.63′′), which belongs to the rural area of the municipality of Barbalha-Ceará. This community is part of the Environmental Protection Area (APA) of the Chapada do Araripe, which is located around the Araripe National Forest (FLONA) [8]. The FLONA includes areas of Cerrado, Carrasco, and Semidecidual Seasonal Forest, locally called wet forest [16].

The community of Macaúba (with 250 families) accesses both the APA and FLONA resources, especially for the collection of Pequi (*Caryocar coriaceum* Wittm.), Faveira (*Dimorphandra gardneriana* Tul.), Janaguba [*Himatanthus drasticus* (Mart.) Plumel], Barbatimão (*Stryphnodendron rotundifolium* Mart.), and Coco Babaçu (*Attalea speciosa* Mart. ex Spreng) [7, 8, 16]. The people of this community live primarily from the extractivism of FLONA's nontimber forest products, subsistence agriculture, pensions, and government support.

### 2.2. Legal Procedures and Informants Selection

The research was authorized by the Research Ethics Committee that involves human beings, from the Health Sciences Center of the Federal University of Pernambuco (CEP/CCS/UFPE), resolution 196/96, and the System of Authorization and Information on Biodiversity (Sisbio), with the authorization numbers 03363812.6.0000.5207, and 32682-1, respectively.

Initial contact with the Macaúba community was carried out through the Macaúba Rural Women's Association and the local health agency, and the objectives of the research were explained to the leaders. Based on the data provided by the health clinic, a random simple probabilistic sampling was used for the selection of informants [17], in which 152 out of the 250 families registered in the community were selected for the interviews.

### 2.3. Data Collection and Processing

Among the 152 families drawn, only 127 people were interviewed (73 women and 54 men), because in residences with two families (11), it was decided to interview only the representative of one of them. In addition, five families were traveling at the time of the interview and nine refused to participate in the study. The interviews were conducted only with the head of each family, which could be both men and women. However, only the one that was present at the residence at the time of the interview was interviewed.

Initially, the socioeconomic data of each informant, such as age, gender, and profession, were recorded. In order to collect information about the local knowledge on known and used plants, free lists were performed [17], through which informants were invited to list the known medicinal plants. For each registered plant the following questions were raised: has this plant been actually used? What are its medicinal uses? Which parts of the plants are used?

Guided tours were conducted for the collection and taxonomic identification of the plants cited by the informants [17], in which the respondents confirmed their vernacular names. The botanical collections were conducted during May and June 2012 and supplemented with collections from our team (Laboratory of Applied and Theoretical Ethnobiology) between the years 2012 and 2013. All the specimens were identified (according to APG [Angiosperm Phylogeny Group] III) and deposited at the Dárdano de Andrade and Lima Herbarium (Herbário Caririense Dárdano de Andrade e Lima, HCDAL) of the Regional University of Cariri (Universidade Regional do Cariri, URCA) in the city of Crato, Ceará. The voucher numbers run from HCDAL 6601 to 6701, 8104 to 8110, and 51592 to 51754. Duplicates were deposited at Professor Vasconcelos Sobrinho herbarium (PEUFR) of the Federal Rural University of Pernambuco (Universidade Federal Rural de Pernambuco- UFRPE), Recife, Pernambuco. Two different methodologies were used to survey priority species for conservation. The first was based on a participatory workshop held with local experts and the second was based on the conservation priority (CP) calculation. The determination of the local experts was performed through the quartile analysis, using the number of citations of medicinal plant uses per informant, through the program BioEstat 5.0 [18]. For this, the result of the third quartile was set as a threshold, which presented 19.5 citations of plant uses. Thus, thirty-two informants were invited to participate in the workshop; however, only eight (five men and three women over 38 years old) were present.

Based on the perception of local experts, data were collected about the environmental availability and intensity of collection of the ten most prominent woody medicinal species. For the selection of these species, a salience analysis was performed with all the species that obtained above 15% of citation (34 species) (Table 1) using the ANTHROPAC 4.0 software [19]. This analysis considered the frequency of citation that a species obtained and its average position in the lists. After that, the ten most salient and arboreal species were selected, which were native to the region.

Two participatory methodologies were used during the participatory workshop: four-cell and classification matrix [20]. In the “four-cell” methodology, the informants classified the species in the following categories: (1) plants with high availability and low collection intensity; (2) plants with high availability and high collection intensity; (3) plants with low availability and low collection intensity; (4) plants with low availability and high collection intensity. Through the “classification matrix” the informants by consensus identified the collection sites of the ten most salient species.

In the second moment, the CP was calculated. For this, the following information was identified for each species: relative density in the collection area, the risk of collection represented by the part of the plant that is collected, the local importance represented by the percentage of citation of the informants, and lastly its diversity of use. In order to obtain the relative density of the species, phytosociological sampling was executed in a Cerrado area, which was indicated by the informants as an important site for the collection of medicinal resources in the region. The total area sampled was 0.5 hectare, distributed within 50 plots of 10 x 10 m. All living woody individuals, with soil diameter at or above 3 cm, were identified and measured on their DNS. With the exception of the relative density data, the other information was obtained through the interviews.

### 2.4. Data Analysis

The normality of the data was verified using the Lilliefors test. In order to verify differences between the total of known and used plants, the Wilcoxon test was used. The possible correlation of the variable age and the number of known medicinal plants was evaluated through the Spearman Correlation test. The ages of the informants were grouped into six classes with intervals of 9 years, having in the first class informants aged 21 years and, in the latter, informants older than 71 years (Table 2). In order to identify if there were differences in local knowledge according to gender and age, Kruskal-Wallis and Student-Newman-Keuls post hoc tests were performed at 5% probability. The Mann-Whitney* U* test was used to analyze whether there were differences in local knowledge as a function of the professional activity. The professions of the informants were grouped into two categories: farmers and nonfarmers. In the farmer category all those who currently practiced or has practiced some activity related to agriculture. In the nonfarmer category, the informants who did not engage in agriculture-related activities were included, such as production assistant, receptionist, public official, school cooks, and assistant of general services. Statistical analyses were performed using the BioEstat 5.0 program [18].

In order to obtain the order of conservation priority (PC) among the ten most prominent medicinal woody species, the formula initially proposed by Dzerefos and Witkowski [21] and adapted by Albuquerque et al. [22] was applied:

(1) $$\begin{array}{c} CP=0.5\left(\;BS\;\right)+0.5\left(\;UR\;\right) \end{array}$$

where BS is biological score and UR is utilization risk

**Step 1 .**

BS= Dx10 (score for the relative density, as in Table 3).

**Step 2 .**

UR= 0.5 (H) + 0.5 (U) x 10

  H = risk of collection score (Table 3).
  D = relative density score of the species in the conforming area (Table 3).
  U = is defined by the average of the local importance sum (L) and the diversity of uses (V) (Table 3).

The collection risk (H) considered the part of the plant that was used. For those species that had more than one part used, the part of the vegetable that had the highest number of citations by the informants was chosen and, consequently, the corresponding score was adopted (Table 3).

The relative density (DR) of the species was calculated by the formula: DR = 100 (Ni/N), where N is the total number of individuals in the sample and Ni is the number of individuals of a particular species in the sample [23]. The local importance (L) refers to the percentage of informants who cited a particular species as medicinal and the diversity of uses (V) refers to the number of different uses that a given species received. The data needed to calculate the value of use (U) and the other parameters for the risk of use were obtained from semistructured interviews and free lists.

For the medicinal species that, according to the expert informants, also had destructive use, such as timber use, the variable wood use (WU) was added to the equation, adding 10 points for these species. Thus, the new equation for the calculation of conservation priority (CP) was CP = 0.5 (BS) + 0.5 (UR) + (WU).

The results of the CP were used to classify the species into risk categories. Category 1: CP> 80, covered priority species with controlled and monitored extraction. Category 2: CP> 60 <80, included the species that present monitored collection and associated with a specific study on the sustainability of the exploitation. Category 3: CP <60, included species suitable for more intensive extraction for medicinal purposes.

## 3. Results

### 3.1. Local Therapeutic Repertoire: Diversity of Known and Used Species

A total of 214 ethnospecies belonging to the therapeutic repertoire of the Macaúba community were registered, of which 167 species were identified and distributed among 140 genera and 67 families (Table 4). The families that presented the highest numbers of medicinal species were as follows: Fabaceae (22 spp.), Asteraceae (12 spp.), and Lamiaceae (10 spp.), with the use of bark being the most prominent (Table 4).

We verified the existence of a significant difference between the number of known plants and those that are effectively used by the informants (Z (U) = 8.72, p <0.01), showing that the repertoire of known plants was much larger than that of plants that are actually used. Most informants (92%) used more than 50% of the ethnospecies they knew. On average, the informants knew 14.84 ± 10.67 and used 11.92 ± 9.62 ethnospecies.

### 3.2. Influence of Gender, Age, and Professional Activity on the Knowledge of Medicinal Plants

The number of medicinal plants cited by farmers and nonfarmers differed significantly between them (Z (U) = 2.23, p = 0.013), with the first presenting greater knowledge about medicinal plants. However, the gender interfered with this knowledge, as men farmers were more knowledgeable than the nonfarmers (Z (U) = 2.61, p = 0.004). Such a difference was not observed among women farmers and nonfarmers (Z U) = 0.17, p = 0.864).

The age of the informant also had an influence on the number of medicinal plants mentioned, with a positive correlation (rs = 0.33, p <0.01). However, this influence differed between age classes (H = 19.76, p <0.01), showing that the older ones presented greater knowledge (Table 2). Among the age classes, it was also possible to observe that there was a difference in knowledge only among women (H = 22.54; p = 0.02), since on average older women knew more about medicinal plants (Table 2).

### 3.3. Local Perception and Conservation Priority Calculation (PC)

The ten most salient species evaluated in the participatory workshop were* Himatanthus drasticus* (Mart.) Plumel;* Hymenaea stigonocarpa* Mart. ex Hayne;* Stryphnodendron rotundifolium* Mart.;* Caryocar coriaceum* Wittm.;* Eschweilera blanchetiana* (O.) Berg.;* Bowdichia virgilioides* Kunth;* Astronium fraxinifolium* Schott;* Copaifera langsdorffii* Desf.;* Hancornia speciosa* Gomes; and* Myracrodruon urundeuva* Allemão (Table 5).

According to the classification of the local specialists, only* H. drasticus* and* H. speciosa* deserved special care about their conservation. The species* H. drasticus*, classified with high availability and high intensity according to experts (Table 5), is in the third position in the calculation of conservation priority (CP) (Table 6). Although it was mentioned as abundant by the specialists, its relative density in the plots was very low, presenting the highest score (10). In turn,* H. speciosa* presented low availability and high intensity of collection, according to the experts' perception, and although this information was confirmed in the CP, it appeared in the fifth position.

The species that appeared in the first and second positions in the CP were* E. blanchetiana* and* M. urundeuva*, although both were classified by local experts as presenting low risk of extinction, low availability, and low collection intensity. The other species were classified as having high availability and low collection intensity (*C. langsdorffii, C. coriaceum, S. rotundifolium, B. virgilioides, A. fraxinifolium *and* H. stigonocarpa*) (Table 5). Although these species were classified as very available, the relative densities of the species used in the CP were very low, following the same score of the species that was in the first position.

According to the CP, seven of the ten medicinal species analyzed (*E. blanchetiana, H. drasticus, H. stigonocarpa, H. speciosa, B. virgilioides, C. langsdorffii, *and* A. fraxinifolium*) would fall into Category 2. These are species that can continue to be collected, provided they are monitored and associated with a specific study on the sustainability of the exploitation. However, four of these species, classified in Category 2, did not present individuals in the sample plots (*H. stigonocarpa, E. blanchetiana, M. urundeuva, *and* A. fraxinifolium*) (Table 6). The other species (*C. coriaceum *and* S. rotundifolium*) were classified in Category 3 and can be considered as suitable species for a more intensive collection for medicinal purposes in the region.

## 4. Discussion

### 4.1. Knowledge and Local Use of Medicinal Plants

In the Araripe region, several studies have reported the occurrence of plant species used for various medicinal products by populations living in the vicinity of the protection area [7–9]. The richness of the ethnospecies cited is high (222) and 50% of them present arboreal habit [9], which was also confirmed in the present study.

The emphasis on the use of medicinal resources from trees suggests that much of the local therapeutic repertoire is based on plants that have a longer life cycle and that, depending on the part accessed, may show a delay in time to express the deleterious effects of some of the collection practices. However, the fact that tree species are most frequently cited is not enough to assert that these species are the most exploited, since not every known species is necessarily used by the informant and, even when they are, the frequency may be low, presenting no risk to conservation.

The argument above is reinforced by the fact that a large part of the informants of the Macaúba community used slightly more than half of what was known, a fact that has also been evidenced by other studies [2, 8]. Although sharing information about medicinal resources favors the uniformity of knowledge among members of a community, the use of a particular resource depends on other factors, such as disease occurrence, people preference, availability, and accessibility of the resource. Thus, knowing the application of a resource does not necessarily imply its use [1, 7, 9].

In the FLONA, plant bark was widely cited as used to treat infections and inflammations, following what has already been indicated in several studies [7, 8, 24–29]. The use of bark is perhaps due to the fact that some species are rich in tannins, secondary compounds that are very effective in curing various diseases [30]. Besides that, these resources do not disappear during the dry season, especially in the Cerrado and Carrasco areas in the FLONA, during which many species lose their leaves.

In some cases, it is not the bark that is used by the extractors, but rather the latex. However, it is necessary to remove the bark to obtain of this resource, as has been reported for* H. speciosa *and* H. drasticus *and* Himatanthus drasticus* in the Araripe region. Baldauf and Santos [16] point out that the increase in the commercialization of bark, which is motivated by the discovery of its efficiency in treating some diseases, can generate negative impacts on plant populations. Borges Filho and Felfili [31] found that the removal of the bark has the potential to cause negative impacts, since it can reach the phloem tissues by interfering with the transport of nutrients and, consequently, affect the vegetative and reproductive growth of the species. In addition, the bark protects individuals against attacks of microorganisms. When large amounts of bark are exploited in the same individual, it can not resist damage and die [32].

The fruits of some species are medicinal resources that are substantially collected in the FLONA. 82% of the uses of* Caryocar coriaceum *(pequi) are indicated as medicinal in the Araripe region, with the exception of pulp and chestnut oil extraction. Many local populations set up camp around FLONA at the time of the pequi crop for the collection and manufacture of oil, using wood from different forest species for cooking the fruits [9]. Thus, the medicinal use of pequi together with its cultural food use [9] reduces the contribution of diaspores that reach the soil to renew the population of the species, which may lead to problems with the dynamics of renewal of the* C. coriaceum* population [25]. This may have social implications to the sustainability of cultural practices in the region.

*Influence of Socioeconomic Factors on Local Knowledge on Medicinal Plants*. Ethnobiological studies that seek to assess the influence of socioeconomic factors under local knowledge are very frequent [7, 33, 34]. Although this influence is not always confirmed, there is evidence that socioeconomic factors can influence knowledge at different scales.

Professional activity was one of the factors that influenced people's knowledge about the uses of medicinal species in the Macaúba community. The fact that the farmers present greater knowledge about medicinal plants indicates that this practice can induce different experiences with the medicinal resource. Most of the farmers also extract the forest products to supplement their incomes, thus promoting knowledge about medicinal plants. However, this can also be the result of the different functions that are exercised by nonfarmers, which are limited to activities very distant from contact with nature, such as production assistant, receptionist, school cooks, and general services assistant. According to Penna and Lamano Ferreira [35], in modern society local knowledge is being altered and adapted to the new socioeconomic demands due to the changes imposed by urbanization and economic development, resulting in changes and acquisition of new values. Another factor is the time that people would have available to have contact with these resources, based on the large number of hours of work required by these nonrural activities, resulting in less contact with natural resources.

Although the knowledge presented a difference between the professional activities, when this knowledge between the genders was analyzed, the women did not follow this pattern. This finding is different from what is found in the literature, which records that women know more about medicinal plants than men, regardless of their collection sites [5, 6]. The prominence of the male farmers of the Macaíba community in the knowledge about medicinal plants may be related to the fact that they go into the forest to extract the forest products. The absence of a difference in knowledge between women farmers and nonfarmers is perhaps justified by the fact that women also have the responsibility of domestic care. Therefore, they have greater contact with the medicinal plants cultivated, which is mostly formed by herbaceous plants, as has been reported by Lozano et al. [9] for the FLONA Araripe region.

The positive influence of age on the knowledge of medicinal plants that we detected in our research was also recorded in several studies [10, 11, 14, 36]. Such a finding was expected, as older people are likely to have more time to accumulate life-long experiences. Although the homogeneity of knowledge is easier to observe when analyzing a species of great cultural importance [7], our study revealed that the homogeneity of knowledge of medicinal species may occur and depends on the interaction of the socioeconomic characteristics of the population, since the knowledge of older farmers women did not differ from that of nonagricultural women, a different pattern from that observed for men.

Undoubtedly, local knowledge about medicinal species is influenced by several factors and represents the accumulation of experiences lived by the person during his life history, in addition to the information that is transmitted to them. This knowledge is of extreme importance for the management and conservation of the resources used, because it reflects the way people classify and perceive the environment. In this way, men can perceive the distribution of resources and their levels of exploitation differently from women. The same can occur between older and younger people, and so on. Thus, access to knowledge and perception can reveal the similarities and disagreements between people's view of reality, providing managers with information on what measures should be taken to succeed in establishing conservation strategies for priority species.

### 4.2. Environmental Perception X Conservation Priority (CP)

Our results show that people living within the same reality may have differing perceptions about the availability of a resource in the environment and the intensity of its exploitation. Although the ten species analyzed in the participatory workshop have appeared numerous times in the free lists, many of them were not perceived as being in evident risk of local extinction, according to the informants' perception. In contrast, species classified as most vulnerable by informants were ranked fifth in the list of priority conservation species obtained by the CP.

The above disagreement between the local perception and conservation priority may be a reflection of the nonupdating of the baseline references, which Pauly [37] called the Baseline Syndrome. According to the author, this syndrome suggests that there are potential limits to the adaptability of traditional knowledge. Even if significant transformations occur in the environment, local populations can maintain behavior and attitudes based on past referrals [38]. The Baseline Syndrome is also considered a sociopsychological phenomenon that describes imprecise human perception of changes in ecosystems, which can have serious implications for species conservation and people's adaptability [39]. Over time, humans modify their notion of healthy ecosystems by adjusting the characteristics of contemporary environments, which may occur due to inefficient intergenerational transmission of knowledge [40], loss of access to traditional resources, transition to market economies, or the influence of modern education [41].

The information that each person acquires or transmits about the environment may be imperfect, generating behavior that is not always correct for the current environment [42]. For example, people may have out-of-date information about the availability and intensity of exploitation of a resource and, therefore, start collecting more intensively. The transmission of this outdated information may lead other people to select the same cultural traits for a compliance bias; that is, people are prone to adopt the most frequent cultural traits in the population, without regard to whether the information is correct or not. Due to this type of bias people can perceive and collect resources based on what is most widespread in the system by other people. This has implications both for people and for plant populations. In the case of people, according to Barkow [43], an unadaptable cultural trait tends to conduct behaviors that may diminish the aptitude of those who accept it. In the case of plant populations, exploitation based on inaccurate or erroneous information may generate a greater pressure of collection, providing negative impacts for the conservation of the resource.

The perception of the informants recorded in this study indicated that people do not associate the problems generated by the collection practices with the local extinction risk of the medicinal resource. In the perception of the informants some plant species does not experience conservation problems because the resource accessed is the bark that is always available in the environment. However, Feitosa et al. [7] evaluated the extractivism of the bark of* S. rotundifolium* Mart. in the same region of the FLONA Araripe and verified the existence of many dead plants, others presenting almost 100% of their bark removed and absence of plants with larger diameters or with intermediate diameters.

Therefore, although the medicinal use of perennial structures favors the continuity of the collection practice [44], the risk of collecting bark in combination with the intensity of collection frequency can generate negative impacts for species [5, 7, 45] and should be cautiously assessed, especially in the case of small populations. According to and Kala [45] and Dhar [46], medicinal species with small populations and experiencing destructive collection are critically endangered in the forests.

It is worth noting that the CP does not distinguish between collection intensity and quantity of resource exploited, assuming that it is related to the part of the plant that is removed. Thus, different parts can cause different negative effects on plants and species that have the barks as extracted resources will probably have a higher priority of conservation by the CP than those that have their leaves collected. This may also create some biases in making decisions about species conservation, since the amount of resource that is collected may directly interfere with the condition of the species being explored.

A person's relationship to the resource can be shaped by their perception. However, the findings of this study showed that there may be a time delay in the perception of the people about the impacts generated on the exploited resource. Perhaps, this occurs simply because of the biological characteristics of the resource and the part of the plant accessed. For example, in the case of pequi, people collect practically all the fruits of the local crop [9], decreasing the number of diaspores in the soil seed bank for population regeneration in FLONA Araripe [9]. However, people do not realize the ecological problem generated because at present the pequi population has a size that meets the local demand of the resource. People's perception should change when the plants reach senility and die, and there are not sufficient pequi regenerators to maintain the collection practice. Thus, this delay may simply represent the time required for the exploited resource to express the deleterious effect of past collections in its population dynamics. Similar fact was detected by Hoffman et al. [47] in the exploitation of* Rhizophora mangle* in Venezuela. There, although the rate of exploitation of this species was high, people did not perceive and continued to exploit it without restrictions.

The high number of species that presented low numbers of individuals in the phytosociological sampling led the CP to group most of the species in Category 2, which means that they have potential to be collected. Among the eight species classified with high values for their density, four (*M. urundeuva, A. fraxinifolium, H. stigonocarpa, *and* E. blanchetiana)* did not present any individuals in the phytosociological sampling performed. This fact does not necessarily indicate that these species have a high conservation priority. The absence of species in phytosociological sampling may reflect a less regular distribution of the plants in the area, rather than low availability or even local nonexistence. In any case, due to their absence in the sampling, the collection should be limited.

## 5. Conclusions

FLONA Araripe proved to be a good source of medicinal resources for the Macaúba community. These resources presented great diversity of habits and origins, with emphasis on native tree plants. However, even in the face of this vast diversity, much of the therapeutic repertoire of this community is composed of species that are not used.

Socioeconomic factors interfered in local knowledge about medicinal plants, which corroborates the evidence found in the literature.

The perception of people when choosing medicinal species with conservation priorities based on their availability in the environment and intensity of exploitation differed from that found by calculating the conservation priority (CP). According to people's perceptions, only* H. speciosa* would have an urgent need of conservation strategies implementation. According to the CP, the species that presented the highest conservation priority was* E. blanchetiana*.

Although the two approaches have presented divergent data, both are important and necessary for decision-making about species that need to receive more conservation attention, as well as for determining sustainable collection strategies. However, we recommend complementary studies to evaluate the sustainability of the extractivism in the region, since some of these species present other types of uses in addition to the medicinal one, which can lead to a greater pressure of use.

## Figures and Tables

**Table 1:** Salience of the medicinal species most cited by informants from the Macaúba community, Barbalha, Ceará, Northeast Brazil (the native tree species used in the “four-cell” tools and “classification matrix” during the participatory workshop with local experts from the Macaúba site are in bold).

**Species**	**Common name**	**Frequency of citation (**%**)**	**Rank average **	**Salience**	**Collection point**
*Plectranthus amboinicus* (Lour.) Spreng.	Malva do reino	52.8	4.6	0.403	Backyard
*Mentha spicata *L.	Hortelã	51.2	6.18	0.368	Backyard
***Myracrodruon urundeuva* Allemão**	Aroeira	47.2	10.23	0.244	local native vegetation
*Lippia alba* (Mill.) N.E.Br. ex. Britton & P.Wilson	Cidreira	45.7	7.41	0.275	Backyard
*Cymbopogon citratus* (DC) Stapf.	Capim santo	37.0	7.23	0.231	Backyard
***Himatanthus drasticus* (Mart.) Plumel**	Janaguba	36.2	7.04	0.24	local native vegetation
*Hymenaea stigonocarpa* Mart. ex Hayne	Jatobá	33.9	10.37	0.199	local native vegetation
*Ruta graveolens *L.	Arruda	33.9	9.65	0.219	Backyard
*Stryphnodendron rotundifolium *Mart.	Barbatimão	30.7	6.51	0.203	local native vegetation
*Citrus sinensis* (L.) Osbeck	Laranja	28.3	12.17	0.13	Backyard
*Chenopodium ambrosioides *L.	Mentruz	28.3	12.22	0.135	Backyard
*Centrosema *sp.	Alcançú	27.6	10.09	0.165	local native vegetation
*Kalanchoe pinnata *(Lam.) Pers.	Mava da Costa	26.8	6.97	0.176	Backyard
*Rosmarinus officinalis *L.	Alecrim	26.8	6.85	0.193	Backyard
*Plectranthus *sp.	Bodo	26.8	7.15	0.172	Backyard
*Eucalyptus citriodora* F. Muell.	Eucalipto	26.8	9.06	0.14	Backyard /local native vegetation
*Croton campestris* A.St.-Hil.	Velame	26.0	8.15	0.159	local native vegetation
***Hancornia speciosa* Gomes**	Mangaba	24.4	9.52	0.15	local native vegetation
**51741**					
***Anacardium occidentale* L.**	Caju	22.8	12.21	0.9	Backyard /local native vegetation
*Egletes viscosa* (L.) Less.	Marcela	21.3	11.07	0.114	Backyard /purchased
*Coutarea hexandra *(Jacq.) K.Schum.	Quina quina	20.5	12.08	0.088	local native vegetation
*Aloe Vera* (L.) Burm. f.	Babosa	20.5	11.0	0.089	Backyard
***Caryocar coriaceum* Wittm.**	Pequi	20.5	10.62	0.092	local native vegetation
*Dorstenia brasiliensis* Lam.	Contra erva	20.5	13.5	0.101	local native vegetation
*Eschweilera blanchetiana* (O. Berg) Miers	Imbiriba	19.7	15.8	0.07	local native vegetation
*Bowdichia virgilioides* Kunth	Sucupira	17.3	6.45	0.127	local native vegetation
*Heliotropium indicum* L.	Crista de Galo	17.3	12.0	0.091	Backyard
*Ocimum gratissimum *L.	Alfavaca	16.5	10.38	0.086	Backyard
*Myristica fragrans* Houtt.	Noz moscada	16.5	18.38	0.057	purchased
***Astronium fraxinifolium* Schott**	Gonçalave	16.5	12.86	0.077	local native vegetation
*Phyllanthus urinaria *L.	Quebra pedra	16.5	12.71	0.083	Backyard
*Copaifera langsdorffii* Desf.	Podoia	15.7	15.1	0.064	local native vegetation
*Ximenia americana *L.	Ameixa	15.0	12.16	0.089	local native vegetation
*Helianthus annuus *L.	Girassol	15.0	16.05	0.065	purchased

**Table 2:** Analysis of variance of the mean distribution of knowledge on medicinal plants by age class and gender of informants from the Macaúba, Barbalha, Ceará, Northeast Brazil site (NI = total informants, SD = standard deviation, NIM = Number of woman informants, and NIH = number of man informants).

**Age class (years)**	**NI**	**Average number of ethnospecies citations**	**Average number of citations**		**NIM**	**NIH**
			**Men**	**Women**		
		***X±*SD**	***X±*SD**	***X±*SD**		
21 – 30	13	7.07*±*4.42A	8.0*±*0Aa	6.8*±*5.7Aa	10	3
31 – 40	15	13.46*±*8.12 AB	13.5*±*5.1Aa	13.5*±*9.18Ba	11	4
41 – 50	22	12.18*±*6.42AB	11.8*±*6.82Aa	12.5*±*6.36Ba	12	10
51 – 60	18	11.88*±*7.63AB	13.3*±*5.98Aa	10.12*±*9.43Ba	8	10
61 – 70	26	19.07*±*11.63B	16*±*6.04Aa	21.71*±*14.79Ba	14	12
>71	33	18.84*±*13.76B	21.1*±*15.36Aa	17.0*±*12.40Ba	18	15

Different capital letters between lines and within the same column, as well as different lowercase letters between columns and within the same row indicate significant differences by Kruskal-Wallis and Student-Newman-Keuls a posteriori at 5%.

**Table 3:** Criteria used for scoring the relative density, collection risks, local importance and diversity of use of medicinal plants (modified by Dzerefos and Witkowski 2001 and Albuquerque et al. 2011a).

**Criterion**	**Score**
**Relative Density (D)**	
None recorded - very low (0 – 1)	10
Low (10 < 3,5)	7
Medium (35 < 7)	4
High (≥ 7)	1

**Collection Risk (H)**	
(i) Destructive plant collection or over-exploitation of roots or bark. The collection involves the removal of the individual.	10
(ii) Aerial structures, such as bark and roots, and removal of parto stem for extraction of latex, which are collected without causing death to the individual.	7
(iii) Permanent aerial structures such as leaves that are removed, potentially affecting plant energy investment, survival and long-term reproductive success.	4
(iv) Removal of transient aerial structure, such as flowers and fruits. Regeneration of the population can be altered in the long term by removal from the seed bank, but the individual plant is not affected.	1

**Local Importance (L)**	
(i) Very High (listed by > de 75% of local informants).	10
(ii) Moderately high (listed by 50-75% of local informants).	7
(iii) Moderately low (listed by 25-50% of local informants).	4
(iv) Very low (listed by < 25% of local informants).	1

**Diversity of use (V)**	
(i) One point is added for each medicinal use up to the maximum of 10.	1 – 10
**(E) Associated Timber Use**	
For species with timber use 10 points are added to the formula	10

**Table 4:** Medicinal plants mentioned in the free list in Macaúba community, Barbalha, Ceará with their respective common name, habit, origin, used part, and uses (plants not found in guided tour; plants brought in from other regions or purchased).

**Family/Scientific name**	**Ethnospecies**	**Habit**	**Origin**	**Used part **	**Use**
**Amaranthaceae**					
*Chenopodium ambrosioides* L.	Mentruz	Herb	Exotic	Leaf	Pain, healing, influenza, verme.
**Amaryllidaceae**					
*Allium sativum* L.	Alho*∗∗*	Herb	Exotic	Bulb, leaf	Heart, fever, gases, influenza.
*Allium cepa* L.	Cebola branca*∗∗*	Herb	Exotic	Bulb	Fever, gases, influenza, cough.
**Anacardiaceae**					
*Anacardium humile* A.St.-Hil.	Cajuí	Tree	Native	Bark	Healing, influenza.
*Anacardium occidentale* L.	Caju	Tree	Native	Bark, weaves, leaf	Healing, inflammation, vaginal, inflammation, influenza.
*Astronium fraxinifolium* Schott	Gonçalave	Tree	Native	Bark, weaves	Bronquitis, catarrh, healing, vaginal discharge, sore throat, influenza, cough.
*Mangifera indica* L.	Manga	Tree	Exotic	Leaf	Influenza.
*Myracrodruon urundeuva* Allemão	Aroeira	Tree	Native	Bark, bark of fruit, weaves	Bronquitis, healing, vaginal discharge, diabetis, catarrh, sour throat, spinal pain, stomachache, gastritis, infuenza, inflammation of woman, cough.
*Spondias purpurea* L.	Siriguela	Tree	Exotic	Leaf	Indigestion, diarrhea, constipation.
**Annonaceae**					
*Annona coriacea* Mart.	Araticum	Tree	Native	Fruit, root, seed	Strengthen bones, animal louse.
					
*Annona muricata* L.	Graviola	Tree	Exotic	Leaf	Cancer, high pressure.
**Apiaceae**					
*Anethum graveolens* L.	Endro*∗∗*	Herb	Exotic	Leaf, seed	Anemia, nausea, child colic, dysentery, Stroke, headache, bellyache, fever.
*Coriandrum sativum* L.	Coentro*∗∗*	Herb	Exotic	Leaf	Bellyache.
*Pimpinella anisum* L.	Erva-doce*∗∗*	Herb	Exotic	Leaf, seed	Ansia, soothing, child colic, dysentery, bellyache, headache, constipation, nerves, cough.
**Apocynaceae**					
*Hancornia speciosa* Gomes	Mangaba	Tree	Native	Bark, leaf, latex	Stomach stuff, cancer, healing, cholesterol, diabetes, fracture, gastritis, hernia, inflammation, broken bone, blow, high pressure, prostate, ulcer, varicose veins.
*Himatanthus drasticus* (Mart.) Plumel	Janaguba	Tree	Native	Latex	Open your appetite, anemia, asthma, heartburn, Stomach stuff, bronchitis, cancer, catarrh, healing, bellyache, stomachache, fracture, gastritis, swelling, inflammation, liver problems, stomach problems, prostate, rheumatism, cough, ulcer, vesicle.
**Arecaceae**					
*Acrocomia aculeata* (Jacq.) Lodd.	Macaúba	Tree	Native	Leaf, fruit	Depression, head wound, high pressure, nerves, cough.
*Cocos nucifera* L.	Coco-da-praia	Tree	Exotic	Bark of fruit, fruit	Swelling, weakness.
*Syagrus cearencis* Noblik	Coco-catolé*∗∗*	Tree	Native	Fruit, root	blindness, eye wound.
**Aristolochiaceae**					
*Aristolochia* sp.	Jarrinha	Creeper	Native	Rhizome, leaf, root	Influenza, tune the blood, epilepsy, cough, healing, fall in hair, fever.
**Asteraceae**					
*Acanthospermum hispidum* DC.	Espinho-de-cigano/ Arritirante	Herb	Native	Leaf, root	Influenza, hepatitis.
*Acmella oleracea* (L.) R.K.Jansen	Agrião*∗∗*	Herb	Native	All the plant	Back pain.
*Ageratum conyzoides* L.	Mentrasto	Herb	Native	All the plant	Colic.
*Artemisia absinthium* L.	Lorma	Herb	Exotic	Leaf	Dor de barriga.
*Artemisia vulgaris* L.	Anador*∗∗*	Herb	Exotic	Leaf	Colic, bellyache, headache, body ache, fever, influenza.
*Bidens pilosa* L.	Espinho-de-agulha carrapicho-de-agulha/picão	Herb	Native	Leaf	Hepatitis.
*Centratherum punctatum* Cass.	Perpeta	Herb	Native	Flower	Tune the blood, leg wounds
*Egletes viscosa* (L.) Less.	Macela*∗∗*	Herb	Native	Flower, fruit, seed	Swollen belly, colic, indigestion, bellyache, gastritis, liver problem.
*Helianthus annuus* L.	Girassol*∗∗*	Herb	Exotic	Seed	Tune the blood, Stroke, indigestion, headache, migraine, fever, thrombosis.
*Matricaria recutita* L.	Camomila*∗∗*	Herb	Exotic	Leaf, flower, seed	Soothing, insomnia.
*Tanacetum vulgare* L.	Pruma*∗∗*	Herb	Exotic	Leaf	Bellyache.
**Bignoniaceae**					
*Crescentia cujete* L.	Coité*∗∗*	Tree	Exotic	Leaf	Kidneys.
*Handroanthus impetiginosus* (Mart. DC.) Mattos	Pau-darco-roxo	Tree	Native	Leaf	Back pain, inflammation, Sore throat.
*Jacaranda brasiliana* (Lam.) Pers.	Caroba	Tree	Native	Root	Tune the blood.
**Bixaceae**					
*Bixa orellana* L.	Urucum	Tree	Native	Bark of fruit, leaf, seed	Catarrh, cholesterol, Influenza, stone in the liver.
**Boraginaceae**					
*Heliotropium indicum* L.	Crista-de-galo	Herb	Native	Leaf, root	Stroke, heart, bellyache, headache, spinal pain, join pain, avoid cancer, fever, fever of child, influenza, dizziness, eye pain.
**Brassicaceae**					
*Brassica rapa* L.	Mostarda*∗∗*	Herb	Exotic	Seed	Stroke, indigestion, constipation, headache, avoid swoon, girth, dizziness, thrombose.
**Bromeliaceae**					
*Ananas sativus *Schult. &Schult. f.	Abacaxi*∗∗*	Herb	Native	Fruit	Lose weight, digestion.
**Cactaceae**					
*Cereus jamacaru* DC.	Mandacarú*∗∗*	Tree	Native	Bark, root	Tune the blood, Kidney stone.
*Opuntia ficus-indica* (L.) Mill.	Palma*∗*	Shrub	Exotic	Bark, leaf	Bronchitis, fatigue.
**Capparaceae**					
*Cleome spinosa* L.	Mussambê	Shrub	Native	Root	Bronchitis, catarrh, influenza, cough, tuberculosis.
**Caprifoliaceae**					
*Sambucus australis* Cham. & Schltdl.	Sabugueiro*∗*	Shrub	Exotic	Flower, leaf	Measles
**Caricaceae**					
*Carica papaya* L.	Mamão*∗*	Tree	Exotic	Leaf, fruit	Indigestion, disentery, digestion, bellyache, constipation.
**Caryocaraceae**					
*Caryocar coriaceum* Wittm.	Pequi	Tree	Native	Leaf, fruit	Bronchitis, fatigue, lump, catarrh, healing, headache, tootache, sore throat, join pain, mouth sore, sorethroat, influenza, broken bone, rheumatism, cough.
**Celastraceae**					
*Maytenus distichophylla* Mart.	Bom-nome*∗*	Tree	Native	Bark	Do not know.
**Chrysobalanaceae**					
*Hirtella* sp.	Caninana	Tree	Native	Bark, liana, root	Headache, spinal pain, rheumatism.
**Convolvulaceae**					
*Operculina* sp.	Batata-de-tiú*∗∗*	Liana	Native	Rhizome	Open animal appetite, tune the blood, healing, headache, fever, influenza, snake bite.
*Operculina macrocarpa *(L.) Urb.	Batata-de-purga*∗∗*	Liana	Native	Rhizome	Kidneys.
**Crassulaceae**					
*Bryophyllum pinnatum *(Lam). Oken	Malva-da-costa/Malva-coronha/ Pabulagem	Herb	Exotic	Bark, leaf, root	Allergy to the skin, lump, healing, indigestion, vaginal discharge, bellyache, headache, sore throat, gases, gastritis, influenza, swelling, inflammation, constipation, cough.
**Cucurbitaceae**					
*Citrullus lanataus* (Thunb.) Matsum. & Nakai	Melancia*∗*	Herb	Exotic	Leaf, fruit, seed	Headache, fever, high pressure.
*Luffa operculata *(L.) Cogn.	Cabacinha*∗∗*	Creeper	Native	Leaf, fruit	Sinusitis.
*Sechium edule* (Jacq.) Sw.	Chuchu*∗∗*	Creeper	Exotic	Leaf	High pressure
**Erythroxylaceae**					
*Erythroxylum ampliofolium* (Mart.) O.E. Schulz	Catuaba	Shrub	Native	Bark, latex, Bark	Aphrodisac, sore throat, body ache, weakness, impotence, nerves, prestate, viagra.
**Euphorbiaceae**					
*Croton blanchetianus* Baill.	Marmeleiro	Shrub	Native	Bark, leaf	Indigestion, bellyache.
*Croton campestris* A.St.-Hil.	Velame	Shrub	Native	Leaf, branch, milk, root	Tune the blood, bronquitis, lump, healing, indigestion, constipation, bellyache, headache, toothache sore throat, earache, fever, influenza, inflammation, broken bone, rheumatism, bad blood, cough.
*Jatropha gossypiifolia* L.	Pinhão-roxo	Shrub	Native	Leaf, latex, All the plant, seed	Stroke, eye disease, headache, tootache, avoid evil eye, disturbed judgment.
*Jatropha mollissima* (Pohl) Baill.	Pinhão-manso	Shrub	Native	Seed	Stroke
*Ricinus communis* L.	Mamona*∗*	Shrub	Exotic	Leaf, seed	Open your appetite, cataract, blindness, bellyache, headache, swelling, drowsiness, swollen chin, dizziness.
**Fabaceae**					
*Amburana cearenses* (Allemão) A.C.Sm.	Imburana/ Imburana-de-cheiro*∗∗*	Tree	Native	Bark	Accelerates chidbirth, healing, woman's disease, back pain, joint pain, fever, influenza, inflammatin, cold, sinusitis, cough.
*Anadenanthera colubrina* (Vell.) Brenan	Angico*∗∗*	Tree	Native	Bark, weaves, woody.	Bronquitis, healing, bellyache, injury, gastritis, influenza, inflammation, lung, burn, cough.
*Bauhinia cheilantha* (Bong.) Steud.	Pata-de-vaca/ Mororó*∗*	Tree	Native	Bark, weaves, leaf	Cholesterol, diarrhea, diabetis, pain when urinating, pain bone, influeza, nerves.
*Bowdichia virgilioides* Kunth	Sucupira/ Sicupira	Tree	Native	Bark, weaves, root, seed	Heartburn, healing, cholesterol, diabetis, bellyache, back pain, pain bone, edema, gastritis, influenza, swelling, snake bite, rheumatism, cough.
*Cajanus cajan* (L.) Millsp.	Andú	Shrub	Exotic	Leaf, seed	Diabetis, bellyache, high pressure.
*Centrosema* sp.	Alcançu	Herb	Native	Root	Bronquitis, fatigue, catarrh, sore throat, influenza, liver problem, cough.
*Copaifera langsdorffii* Desf.	Podoia/ Copaíba	Tree	Native	Bark, leaf, latex, oil, seed	Healing, indigestion, Stroke, headache, back pain, migraine, gastritis, inflammation, lung, nerves, intestine, rheumatism, sinusitis, dizziness.
*Dimorphandra gardneriana* Tul.	Faveira	Tree	Native	Bark, leaf, fruit, latex, root	Snake bite.
*Dioclea grandiflora* Benth.	Mucunã	Creeper	Native	Bark, leaf	Influenza.
*Enterolobium contortisiliquum* (Vell.) Morong	Tamburí*∗*	Tree	Native	Bark	Swelling.
*Hymenaea* sp.	Jatubí*∗*	Tree	Native	Do not know	Do not know
*Hymenaea stigonocarpa* Mart. ex Hayne	Jatobá	Tree	Native	Bark, weaves, leaf	Tune the blood, bronquitis, tiredness, catarrh, itchiness, sore throat, influenza, inflammation, body drowsiness, hoarseness, cough.
*Leucaena leucocephala* (Lam.) de Wit	Linhaça*∗∗*	Tree	Exotic	Seed	Inflammation of the uterus.
*Libidibia férrea* (Mart. ex Tul.) L.P.Queiroz	Pau-ferro	Tree	Native	Bark, weaves, fruit	Bronquitis, healing, depression, fever, influenza, inflammation of woman, nerves, cough.
*Macroptilium bracteatum* (Nees & C. Mart.) Maréchal & Baudet	Flor-de- mulher	Herb	Native	All the plant	sore throat.
*Mimosa tenuiflora* (Willd.) Poir.	Jurema preta	Tree	Native	Bark, weaves, leaf, root	Healing, diarrhea, bellyache, tootache, injury, influenza, inflammation, inflammation of woman.
*Mimosa pudica* L.	Malícia	Shrub	Native	Leaf, root	High pressure.
*Peltophorum* sp.	Canafistula*∗∗*	Tree	Native	Leaf	Tune the hair.
*Poincianella pyramidalis* (Tul.) L.P.Queiroz	Catingueira*∗*	Tree	Native	Bark, flower	Headache.
					
*Senna occidentalis* (L.) Link	Manjerioba	Shrub	Native	Leaf, root, sement	Catarrh, Stroke, bellyache, headache, fever, influenza, cold, cough.
*Stryphnodendron rotundufolium* Mart.	Barbatimão	Shrub	Native	Bark, weaves	Cancer, healing, vaginal discharge, bellyache, injury, gastritis, inflammation, kidneys, sinusitis.
*Tamarindus indica* L.	Tamarindo	Shrub	Exotic	Leaf	Diarrhea, bellyache.
**Krameriaceae**					
*Krameria tomentosa* A. St.-Hil.	Carrapicho-de-boi	Shrub	Native	Root	Anemia, menstruation.
**Lamiaceae**					
*Lavandula* sp	Alfazema	Herb	Exotic	Seed	Bellyache.
*Leonotis nepetifolia* (L.) R. Br..	Cordão-de-São Francisco	Herb	Exotic	Flower	Azia, indigestion.
*Mentha spicata* L.	Hortelã	Herb	Exotic	Leaf, seed	Open your appetite, Stroke, lump, tiredness, itchness, heart, swoon, bellyache, headache, spinal pain, tootache, sore throat, body ache, eye pain, lose weight, migraine, fever, girth, influenza, inflammation of woman, bad breath, cold, blow in the heart, dizziness, cough, thrombosis, vomit.
*Mentha pulegium* L.	Hortelã poejo	Herb	Exotic	Leaf	Headache, migraine, girth, verme.
*Ocimum basilicum* L.	Manjericão	Herb	Exotic	Leaf	Earache, influenza, cough.
*Ocimum gratissimum *L.	Alfavaca	Herb	Exotic	Leaf, all the plant, root, seed	Anemia, cancer, healing, menstrual colic, headache, woman pain, earache, spinal pain, kidney, migraine, inflammation, high pressure, sinusitis.
*Plectranthus amboinicus* (Lour.) Spreng.	Malva-do-reino	Herb	Exotic	Leaf, seed	Open your appetite, burning in the eyes, bronquitis, fatigue, catarrh, healing, colic, indigestion, vaginal discharge, bellyache, headache, sore throat, stanch blood, skin wound, influenza, inflammation, cough.
*Plectranthus barbatus* Andrews	Sete dor	Shrub	Exotic	Leaf	Abortive, swollen belly, indigestion, bellyache, headache, back pain, inflammation of woman, liver problem.
*Plectranthus neochilus* Schltr.	Boldo/ Boldinho/ Boldo da folha mole	Herb	Exotic	Leaf	Open your appetite, heartburn, colic, indigestion, hangover, diarrhea, bellyache, headache, nausea, liver, gastritis, high pressure.
*Rosmarinus officinalis* L.	Alecrim*∗*	Herb	Exotic	Leaf, seed	Fever, headache, constipation, bellyache, stomach problem, colic, cough, heart problem, high pressure.
**Lauraceae **					
*Laurus mobilis* L.	Louro*∗∗*	Herb	Exotic	Leaf	Constipation.
*Cinnamomum* sp.	Canela*∗∗*	Shrub	Exotic	Bark, seed	Weakness, nerves.
*Persea americana* Mill	Abacate*∗*	Tree	Exotic	Leaf, seed	Bellyache, kidney pain, liver.
**Lecythidaceae**					
*Eschweilera blanchetiana* (O. Berg) Miers	Imbiriba	Tree	Native	Bark, bark of fruit, flower, leaf, fruit, seed	Swollen belly, colic, indigestion, bellyache, headache, stomach, cough, vomito.
**Liliaceae**					
*Lilium* L.	Anil estrelado*∗∗*	Herb	Exotic	Flower	Fever.
**Malphigiaceae**					
*Byrsonima sericea* DC.	Muricí vermelho	Arbusto	Native	Weaves	Diabetis.
*Malphigia glabra* L.	Acerola*∗*	Arbusto	Exotic	Leaf	Open your appetite, Tune the blood, Influenza.
**Malvaceae**					
*Gossypium barbadense* L.	Algodão*∗*	Shrub	Exotic	Leaf, seed	Indigestion.
*Pseudobombax marginatum* (A. St.-Hil., Juss. & Cambess.) A. Robyns	Imbiratanha*∗*	Tree	Native	Bark	Diabetis, spinal pain.
*Sida cordifolia* L.	Malva-branca	Herb	Native	Leaf, root	Coceira, vaginal discharge, fever, influenza, inflammation of woman, cough.
*Theobroma cacao* L.	Cacaú*∗∗*	Tree	Exotic	Seed	Dizziness.
**Marantaceae**					
*Maranta arundinacea* Blanco	Araruta*∗*	Herb	Native	Rhizome	Malnutrition.
**Menispermaceae**					
*Cissampelos ovalifolia* DC.	Orelha-de-onça*∗*	Herb	Native	Root, rhizome	Indigestion, influenza, cough.
**Moraceae**					
*Dorstenia brasiliensis* Lam.	Contra-erva	Herb	Native	Leaf, root	Catarrh, diarrhea, fever, influenza, cough.
**Musaceae**					
*Musa paradisiaca* L.	Banana prata*∗*	Herb	Exotic	Fruit	Diarrhea.
**Myristicaceae**					
*Myristica fragrans* Houtt.	Noz moscada*∗∗*	Tree	Exotic	Seed	Ansia, Stroke, colic, indigestion, heart, swoon, bellyache, headache, numbness, nerves, kidneys, dizziness, cough, thrombosis.
**Myrtaceae**					
*Eucalyptus citriodora* F. Muell.	Eucalipto*∗*	Tree	Exotic	Leaf	Fatigue, catarrh, headache, fever, couch, rhinitis, sinusitis, cough.
*Eugenia uniflora L.*	Pitanga*∗*	Shrub	Native	Leaf	Verme, indigestion, diarrhea.
*Myrciaria* sp.	Cambuí*∗*	Shrub	Native	Leaf	Do not know.
*Psidium guajava* L.	Goiaba/ Goiaba branca	Shrub	Native	Leaf	Diarrhea, bellyache, vomito.
*Psidium* sp1	Araçá vermelho	Tree	Native	Leaf	Nerves.
*Psidium* sp2	Araçá branco	Tree	Native	Leaf	Nerves.
*Psidium myrsinites* DC.	Araçá	Tree	Native	Leaf	Bellyache, diarrhea, indigestion, high pressure.
*Psidium* sp4	Araçá amarelo	Tree	Native	Leaf	Nerves.
*Syzygium cumini* (L.) Skeels	Azeitona preta	Tree	Exotic	Flower, seed	Colic, indigestion, fever, influenza, high pressure, cough, vomito.
**Nyctaginaceae**					
*Boerhavia diffusa* L.	Pega-pinto	Herb	Exotic	Root	Allergy, vaginal discharge, inflammation, inflammation of woman.
**Olacaceae**					
*Ximenia americana* L.	Ameixa	Tree	Native	Bark, weaves, fruit	Tune your blood, anemia, healing, diabetis, bellyache, headache, stomach ache, gastritis, inflammation, inflammation of woman.
**Papaveraceae**					
*Argemone mexicana* L	Carro santo	Herb	Exotic	Leaf, root, seed	Stroke, influenza, cough.
**Passifloraceae**					
*Passiflora edulis* Sims	Maracujá*∗*	Creeper	Native	Bark of fruit, leaf, fruit	Soothing, diabetis, insomnia, high pressure.
*Passiflora* cincinnata Mast.	Maracujá do mato	Creeper	Native	Leaf fruit	Nerves, high pressure.
*Turnera subulata* Sm.	Xanana	Herb	Native	Leaf, root	Vaginal discharge, inflamamtion
**Pedaliaceae**					
*Sesamum orientale* L.	Gergilim*∗∗*	Shrub	Exotic	Seed	Stroke, indigestion, bellyache, headache fever.
**Phyllanthaceae**					
*Phyllanthus urinaria* L.	Quebra pedra	Herb	Native	Bark, leaf, all the plant	Indigestion, bellyache kidney pain, fever, broken bone.
**Phytolaccaceae**					
*Petiveria alliacea* L.	Tipí	Herb	Native	Leaf, root	Vaginal discharge, rheumatism, Stroke.
Piperaceae					
*Piper nigrum* L.	Pimenta do reino*∗∗*	Shrub	Exotic	Seed	Migriane.
*Piper aduncum* L.	Pimenta de nico	Shrub	Native	Bark, fruit, seed	Migraine.
**Plantaginaceae**					
*Scoparia dulcis* L.	Bassorinha	Herb	Native	Leaf, root, all the plant	Allergy, chickenpox, headache, fever, influenza.
**Poaceae**					
*Cymbopogon citratus* (DC) Stapf.	Capim santo*∗*	Herb	Exotic	Leaf, root	Open your appetite, colic, bellyache, headache, fever, influenza, nerves, high pressure, cough.
*Pennisetum* sp.	Capim de planta*∗*	Herb	Exotic	Root	Swelling.
*Saccharum officinalis* L.	Cana-de-açúcar*∗*	Herb	Native	Leaf	Back pain, high pressure.
**Polygalaceae**					
*Polygala paniculata* L.	Caninaninha de cipó fino	Shrub	Native	Root	Rheumatism.
**Proteaceae**					
*Roupala montana* Aubl.	Congonha	Tree	Native	Leaf, all the plant	Indigestion, bellyache, nerves.
**Punicaceae**					
*Punica granatum* L.	Romã	Tree	Exotic	Bark the fruit, leaf, fruit	Diarrhea, sore throat.
**Rhamnaceae**					
*Zizyphus joazeiro* Mart.	Juá, juazeiro	Tree	Native	Bark, weaves, leaf	Healing, bellyache, influenza, inflamation, cough.
**Rosaceae**					
*Rosa alba* L.	Rosa branca*∗*	Herb	Exotic	Flower	Inflammation.
*Malus domestica* Borkh.	Maçã*∗∗*	Arbusto	Exotic	Fruit	Lose weight.
**Rubiaceae**					
*Coffea arabica* L.	Café*∗*	Arbusto	Exotic	Seed	Swelling.
*Coutarea hexandra* (Jacq.) K.Schum.	Quina-quina*∗*	Árvore	Native	Bark, leaf	Healing, bellyache, headache, fever, influenza, sinusitis.
*Tocoyena formosa* (Cham. & Schltdl.) K.Schum.	Genipapinho	Shrub	Native	Bark	Broken bone.
**Rutaceae**					
*Citrus aurantifolia* (Christm.) Swingle	Limão azedo*∗*	Tree	Exotic	Fruit	Cataract.
*Citrus* sp1	Laranja da terra*∗*	Tree	Exotic	Bark in the fruit, leaf	Cancer, diarrhea, bellyache, fever, gastritis.
*Citrus limon* (L.)Burm.f.	Limão*∗*	Tree	Exotic	Leaf, fruit	Fever, influenza.
*Citrus* sp3	Lima*∗*	Tree	Exotic	Fruit	Hepatitis.
*Citrus sinensis* (L.) Osbeck	Laranja*∗*	Tree	Exotic	Bark, Bark in the fruit, leaf, fruit	Open your appetite, indigestion, bellyache, headache, nerves, high pressure.
*Citrus* sp4	Lima doce/ Lima de umbigo*∗*	Tree	Exotic	Bark, leaf	Indigestion, migraine, hepatitis, nerves.
*Murraya paniculata* (L.) Jack	Jasmim laranja*∗*	Shrub	Exotic	Leaf	Indigestion.
*Pilocarpus microphyllus* Stapf ex Wardleworth	Jaborandi*∗*	Shrub	Native	Bark, leaf, root	Fever, influenza.
*Ruta graveolens* L.	Arruda	Herb	Exotic	Leaf, all the plant	Bellyache, headache, fever, influenza.
**Sapindaceae**					
*Talisia esculenta* (Cambess.) Radlk.	Pitomba	Tree	Native	Do not know	Do not know.
*Serjania* sp.	Cipó de vaqueiro*∗*	Creeper	Native	Root	Prostate.
**Sapotaceae**					
*Sideroxylon obtusifolium* (Roem. & Schult.) T.D. Penn.	Quixaba*∗*	Shrub	Native	Bark, weaves, leaf	Diabetis, inflammation, broken bone.
**Smilacaceae**					
*Smilax staminea* Griseb.	Japecanga	Creeper	Native	Bark, root	Rheumatism.
**Solanaceae**					
*Capsicum frutescens* L.	Pimenta malagueta*∗∗*	Herb	Exotic	Leaf	Lump.
*Solanum erianthum* D. Don	Jurubeba branca	Herb	Exotic		Do not know.
**Urticaceae**					
*Cecropia* Loefl.	Toré	Tree	Native	Leaf	Cancer, diabetes.
**Verbenaceae**					
*Lippia alba* (Mill.) N.E.Br. ex. Britton & P.Wilson	Cidreira	Shrub	Exotic	Leaf, branch	Open your appetite, colic, indigestion diarrhea, bellyache, headache, influenza, nerves, high pressure, constipation, cough.
**Violaceae**					
*Hybanthus calceolaria* (L.) Oken	Papaconha*∗∗*	Herb	Native	Root	Catarrh, fever, influenza, cough, verme.
**Vochysiaceae**					
*Qualea parviflora* Mart.	Pau piranha/pau terra	Tree	Native	Bark	Cow abortion.
**Xanthorrhoeaceae**					
*Aloe Vera* (L.) Burm. f.	Babosa	Herb	Exotic	Leaf	Open your appetite, bronquitis, cancer, healing, gastritis, influenza, inflamation.
**Zingiberaceae**					
*Alpinia zerumbet* (Pers.) B.L.Burtt & R.M.Sm.	Exprito/ Colônia	Herb	Exotic	Leaf, flower	Headache, influenza, high pressure.
*Zingiber officinale* Roscoe	Gengibre*∗∗*	Herb	Exotic	Rhizome	Cough.

**Table 5:** Results of the four-cell tool performed with the local experts from the Macaúba community, Barbalha, Ceará, Northeast Brazil.

**High environmental availability and low collection intensity**	**High environmental readiness and high collection intensity**
*Copaifera langsdorffii* Desf. *Caryocar coriaceum* Wittm. *Stryphnodendron rotundifolium *Mart. *Bowdichia virgilioides* Kunth *Hymenaea stigonocarpa* Mart. ex Hayne *Astronium fraxinifolium Schott*	*Himatanthus drasticus* **(Mart.) Plumel**

**Low environmental availability and low collection intensity**	**Low environmental availability and high collection intensity**

* Myracrodruon urundeuva* Allemão *Eschweilera blanchetiana* (O. Berg) Miers	*Hancornia speciosa* Gomes

**Table 6:** Conservation priority of the 10 most salient woody species of the Araripe National Forest and Araripe National Forest Environmental Protection Area, Ceará, Northeast Brazil (D = relative density score, DR = relative density, H = risk score, L = local importance score, NI = number of individuals, NU = total number of uses, CP = conservation priority, U = use value, and V = diversity of uses score, *∗* associated logging use).

**Scientific name**	**DR **%	**D**	**H**	**NI**	**L**	**NU**	**V**	**U**	**CP**
*Eschweilera blanchetiana* (O. Berg) Miers*∗*	0	10	1	0	1	11	10	5.5	74.00
*Myracrodruon urundeuva* Allemão	0	10	7	0	4	17	10	7	69.25
*Himatanthus drasticus* (Mart.) Plumel	0.8	10	7	19	4	23	10	7	69.25
*Hymenaea stigonocarpa* Mart. ex Hayne	0	10	7	0	4	13	10	7	69.25
*Hancornia speciosa* Gomes	0.21	10	7	5	1	17	10	5.5	65.50
*Bowdichia virgilioides* Kunth	0.21	10	7	5	1	14	10	5.5	65.50
*Copaifera langsdorffii* Desf.	0.08	10	7	2	1	16	10	5.5	65.50
*Astronium fraxinifolium* Schott	0	10	7	0	1	7	7	4	61.75
*Caryocar coriaceum* Wittm.	1.14	7	1	27	1	19	10	5.5	49.00
*Stryphnodendron rotundifolium *Mart.	5.95	4	7	141	4	11	10	7	39.25

## Data Availability

The data used to support the findings of this study are included within the article.
